# Surface demineralized freeze-dried bone allograft followed by reimplantation in a failed mandibular dental implant

**DOI:** 10.1093/rb/rbad102

**Published:** 2023-11-17

**Authors:** Jing Zhang, Jie Wang, Jiayi You, Xuan Qin, Huimin Chen, Xiantong Hu, Yantao Zhao, Yang Xia

**Affiliations:** Jiangsu Key Laboratory of Oral Diseases, Nanjing Medical University, Nanjing, Jiangsu 210029, PR China; Jiangsu Province Engineering Research Center of Stomatological Translational Medicine, Nanjing Medical University, Nanjing, Jiangsu 210029, PR China; Jiangsu Key Laboratory of Oral Diseases, Nanjing Medical University, Nanjing, Jiangsu 210029, PR China; Jiangsu Province Engineering Research Center of Stomatological Translational Medicine, Nanjing Medical University, Nanjing, Jiangsu 210029, PR China; Jiangsu Key Laboratory of Oral Diseases, Nanjing Medical University, Nanjing, Jiangsu 210029, PR China; Jiangsu Province Engineering Research Center of Stomatological Translational Medicine, Nanjing Medical University, Nanjing, Jiangsu 210029, PR China; Jiangsu Key Laboratory of Oral Diseases, Nanjing Medical University, Nanjing, Jiangsu 210029, PR China; Jiangsu Province Engineering Research Center of Stomatological Translational Medicine, Nanjing Medical University, Nanjing, Jiangsu 210029, PR China; Department of Restorative Dental Sciences, Faculty of Dentistry, The University of Hong Kong, Hong Kong SAR, PR China; Senior Department of Orthopedics, The Fourth Medical Center of PLA General Hospital, Beijing, 100048, PR China; Beijing Engineering Research Center of Orthopedics Implants, Beijing 100048, PR China; State Key Laboratory of Military Stomatology, Xi'an 710032, PR China; Senior Department of Orthopedics, The Fourth Medical Center of PLA General Hospital, Beijing, 100048, PR China; Beijing Engineering Research Center of Orthopedics Implants, Beijing 100048, PR China; State Key Laboratory of Military Stomatology, Xi'an 710032, PR China; Jiangsu Key Laboratory of Oral Diseases, Nanjing Medical University, Nanjing, Jiangsu 210029, PR China; Jiangsu Province Engineering Research Center of Stomatological Translational Medicine, Nanjing Medical University, Nanjing, Jiangsu 210029, PR China

**Keywords:** dental implant, implant failure, allogeneic bone, bone regeneration, reimplantation

## Abstract

The removal of a failed implant with high torque causes significant damage to the surrounding tissue, compromising bone regeneration and subsequent osseointegration in the defect area. Here, we report a case of carrier screw fracture followed by immediate implant removal, bone grafting and delayed reimplantation. A dental implant with a fractured central carrier screw was removed using the bur-forceps technique. The resulting three-wall bone defect was filled with granular surface demineralized freeze-dried bone allograft (SD-FDBA). Cone-beam computerized tomography was performed at 1 week, 6 months and 15 months postoperatively and standardized for quantitative evaluation. The alveolar bone width and height at 15 months post-surgery were about 91% of the original values, with a slightly lower bone density, calculated using the gray value ratio. The graft site was reopened and was found to be completely healed with dense and vascularized bone along with some residual bone graft. Reimplantation followed by restoration was performed 8 months later. The quality of regenerated bone following SD-FDBA grafting was adequate for osseointegration and long-term implant success. The excellent osteogenic properties of SD-FDBA are attributed to its human origin, cortical bone-like structure, partly demineralized surfaces and bone morphogenetic protein-2-containing nature. Further investigation with more cases and longer follow-up was required to confirm the final clinical effect.

## Graphical Abstract

**Figure:**
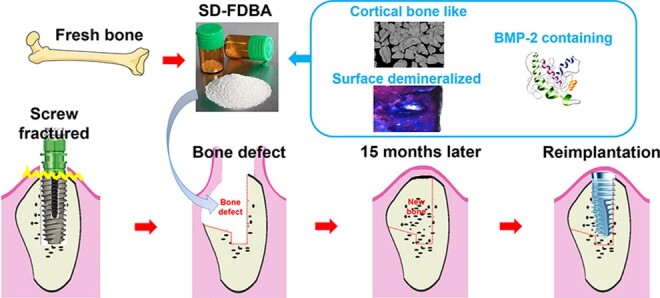

## Introduction

Dentition defects are commonly encountered in dental practice and affect the occlusion, mastication and diet. Fixed, removable and implant dentures are the major treatments for dentition defects. Fixed dentures provide improved fixation, but require healthy surrounding teeth. Preparation of the abutment teeth for fixed dentures requires the removal of the bulk of hard tissue, which increases the risk of pulp exposure and postoperative complications in endodontically treated teeth, and reduces the clinical efficacy of the restoration [1]. Removable dentures are cheaper, minimally invasive and easier to repair or replace. However, they cover the gingival margins and alter the quantity and quality of plaque, compromising oral hygiene. Moreover, they are uncomfortable to wear and only partly restore the masticatory function [2]. In contrast, dental implants provide improved comfort and aesthetics because of the lack of bases and clasps, which may cause a foreign body sensation and affect speech and swallowing. Dental implants can be used to restore the chewing function without damaging the surrounding teeth, which makes them the preferred option for patients with dentition defects [3].

The quality and quantity of alveolar bone dictate the appropriate surgical technique and implant type and are important for primary stability and long-term implant success. Poor bone quality and insufficient quantity can negatively affect implant survival rates. Bone density strongly correlates with dental implant stability [4]; primary stability is difficult to achieve with poor bone density, which then leads to higher implant failure rates. Long-term implant success requires good-quality bone surrounding the implant and a sound interface between them to prevent excessive bone resorption and improve healing. Bone augmentation is commonly performed to enhance the quality and dimensions of the alveolar bone before implantation. Common conditions requiring alveolar bone augmentation include congenital developmental defects, periodontal disease, tooth loss, bone resorption due to infection or inflammation, and trauma [5].

Bone augmentation techniques include extraction socket grafting, horizontal ridge augmentation, vertical ridge augmentation and sinus augmentation, etc. [6]. Bone grafts can also be combined with several adjunctive techniques, including the use of growth and differentiation factors, particulate and block grafting, distraction osteogenesis, guided bone regeneration and alveolar ridge preservation (ARP) [7]. Several graft materials are available, including natural bone grafts, synthetic bone substitutes, osteogenic cell-infused bone substitutes, composite bone substitutes and growth factor-based bone substitutes [8]. The aim of bone grafting is to promote the formation of living and reactive tissues that can undergo sustained remodeling to maintain mechanical and biological functions. Autografts are considered the gold standard for bone augmentation, but their use is limited because of the drawbacks, including donor site morbidity, limited quantity and unpredictable resorption.

Allografts offer an alternative because they possess properties similar to autografts without their drawbacks. Allografts obtained from donors of the same species are osteoinductive and osteoconductive. Sterilization and mechanical debridement of allografts can prevent the transmission of diseases, such as hepatitis and human immunodeficiency virus. They are available in various forms, including cortico-cancellous, cortical and demineralized bone matrix grafts as pieces, chips, wedges, pegs and powders. Commercially available allografts are mostly freeze-dried bone allografts (FDBAs) and demineralized freeze-dried bone allografts (DFDBAs). The processing of FDBA and DFDBA is identical except for demineralization, which is unique to DFDBA. They are both widely applied in dental clinics where bone grafting is needed. Interestingly, it seems that a combination of FDBA and DFDBA is better than either one alone [9, 10].

Therefore, in this study, a surface demineralized FDBA (SD-FDBA) particles were used in a mandibular defect caused by the immediate removal of a screw-fractured dental implant. Bone healing was evaluated using cone-beam computerized tomography (CBCT) at different time points. Reimplantation of the defect was done after primary healing was confirmed through radiographic assessment, and the patient was followed up after restoration. The present case report shows the effects of SD-FDBA on bone regeneration and dimensional preservation without barrier membrane and exogenous bioactive factor in a surgically created three-wall bone socket. The results showed optimal osseointegration of the dental implants with augmented bone and a good long-term clinical prognosis. The reasons for the excellent osteogenic properties of this SD-FDBA were explored from aspects of its structure and composition.

## Materials

The commercial allografts SD-FDBA (Trade name: allogeneic bone transplants) were provided by Xinkangchen Medical Technology Development Co. (Beijing, China). The surface morphology and element composition were analyzed by scanning electron microscope (SEM) with energy dispersive spectroscopy (EDS, S-3400N, Hitachi, Japan). The microstructure and mineralization were explored by a micro-CT SkyScan-1176 (Bruker, Billerica, USA) and Masson staining of undecalcified histological sections. Bone morphogenetic protein (BMP)-2 content was measured by a BMP-2 ELISA Kit (Abcam, UK). The experimental details are provided as Supplementary data.

## Clinical study and results analysis

A female aged 40 years presented to the Stomatological Hospital of Nanjing Medical University for prosthetic restoration of the right lower second premolar (#29). The patient had no pre-existing medical conditions, was a nonsmoker, and wanted restoration of the masticatory function.

**Figure 1.**
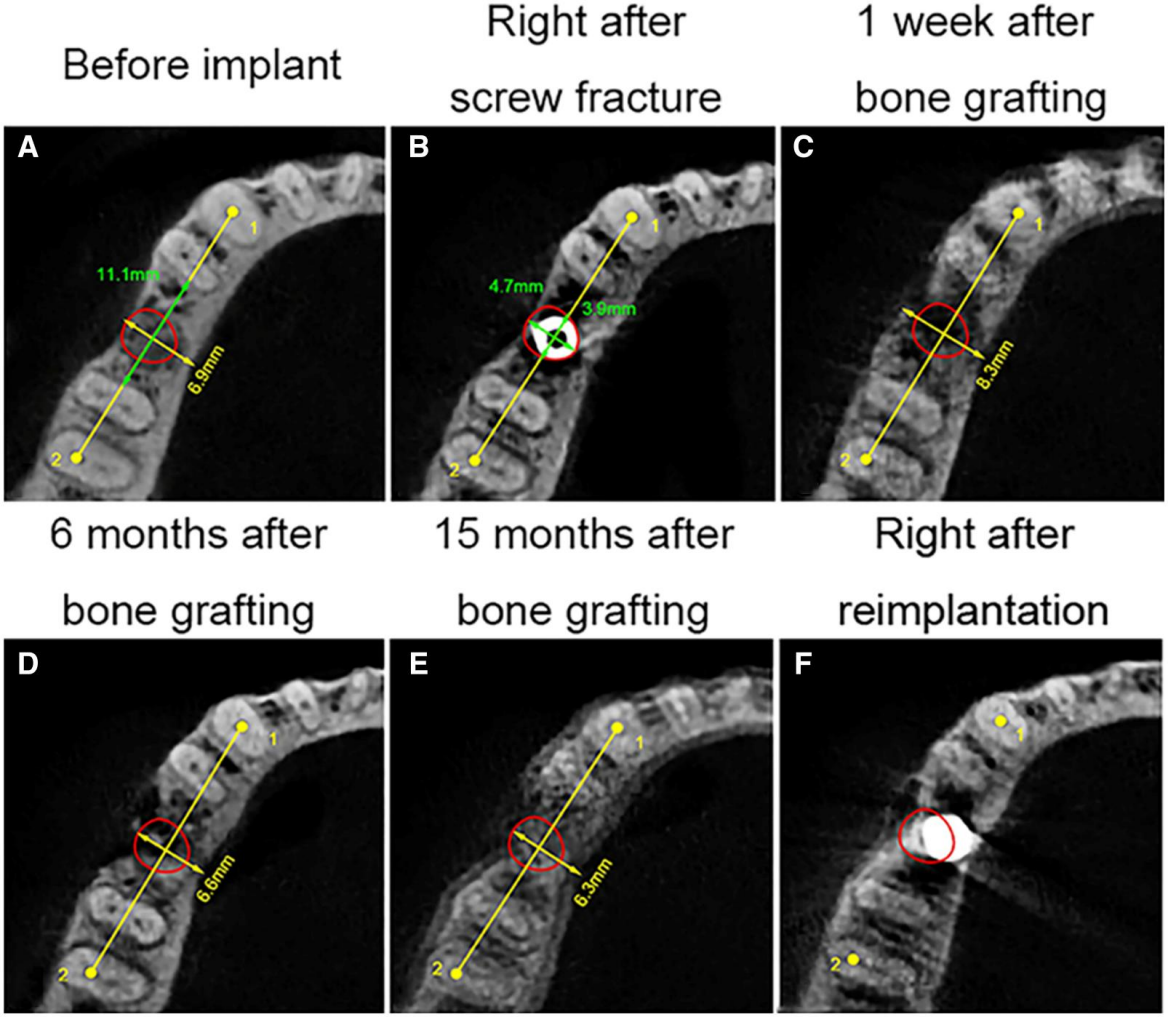
Horizontal CBCT sections at different time points: (**A**) before implant, (**B**) immediately after screw fracture, (**C**) 1 week after bone grafting, (**D**) 6 months after bone grafting, (**E**) 15 months after bone grafting and (**F**) immediately after reimplantation. The red oval shows the bone defect.

**Figure 2.**
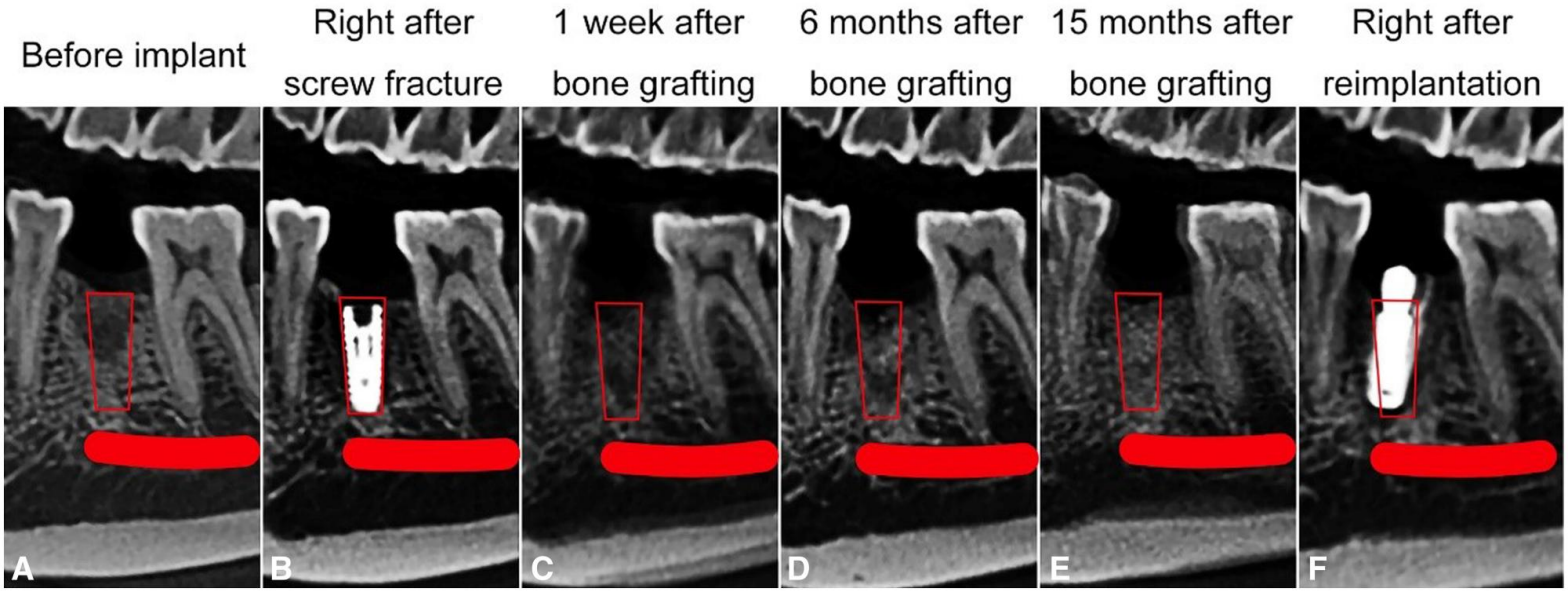
Sagittal CBCT sections at different time points: (**A**) before implant, (**B**) immediately after screw fracture, (**C**) 1 week after bone grafting, (**D**) 6 months after bone grafting, (**E**) 15 months after bone grafting and (**F**) immediately after reimplantation. The red box represents the bone defect, and the red line indicates the mandibular nerve canal.

**Figure 3.**
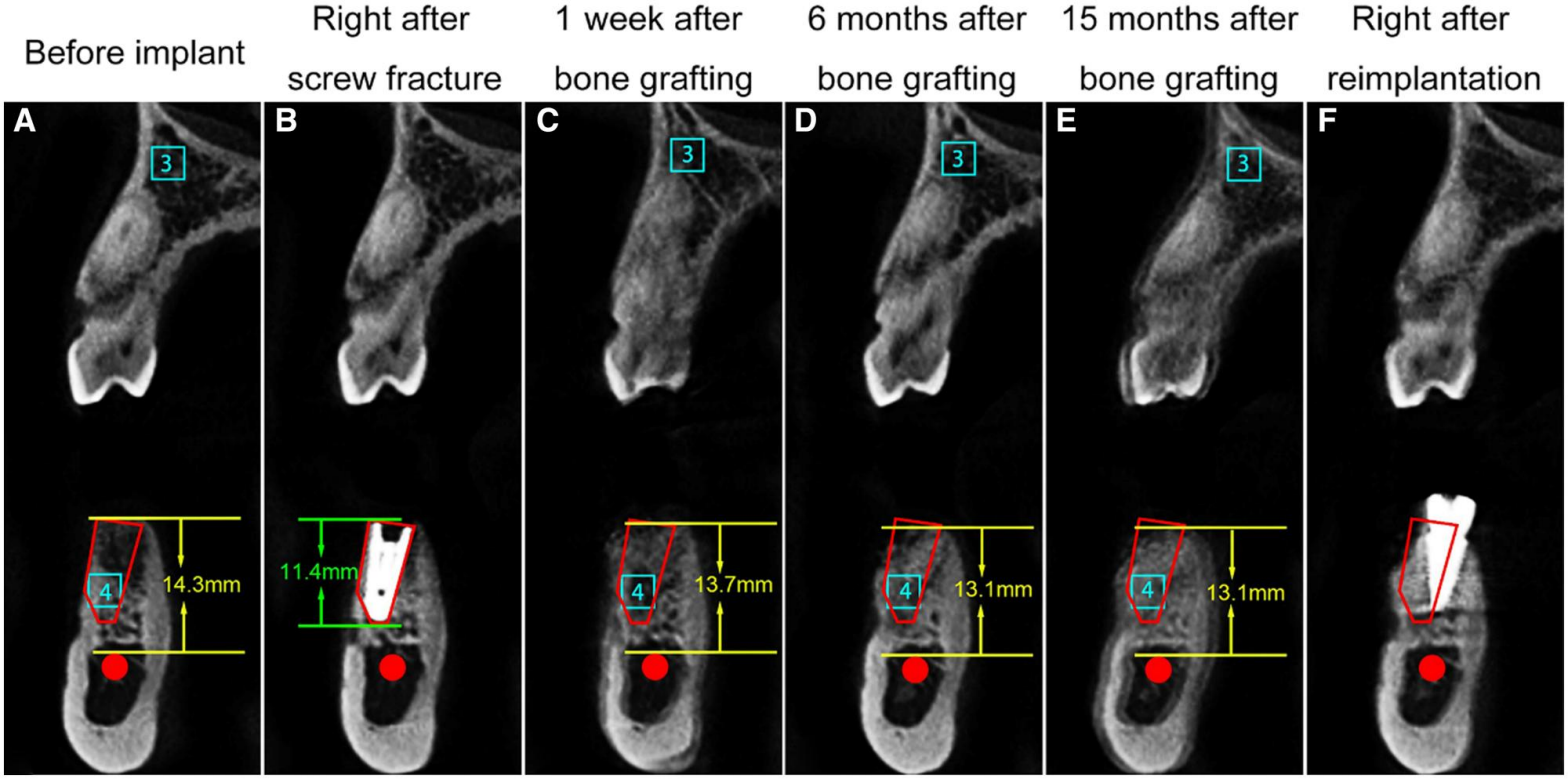
Coronal CBCT sections at different time points: (**A**) before implant, (**B**) immediately after screw fracture, (**C**) 1 week after bone grafting, (**D**) 6 months after bone grafting, (**E**) 15 months after bone grafting and (**F**) immediately after reimplantation. The red box represents the bone defect, and the red dot indicates the mandibular nerve canal.

A complete oral examination and CBCT (Rayscan α-3D, Ray, South Korea) were performed to assess the mandible, including bone mass and positions of the mental foramen and mandibular neural canal. The raw data were reconstructed using the CBCT software (Xelis Dental 1.0.6.2 BN11). The extraction socket of tooth #29 showed appropriate bone healing; it was gray-white on CBCT images but lighter than the surrounding alveolar bone. The distance between teeth #28 and #30 was about 10 mm. The alveolar dimensions (width: the distance between buccal and lingual bone walls; height: the distance from the alveolar crest to the upper wall of the mandibular neural canal) were approximately 6.9 mm × 14.3 mm (Figures 1A–3A). A 3.7 mm × 10 mm Osstem implant (TSIII SA, Republic of Korea) with a central screw for connecting the implant and its carrier was chosen.

A horizontal incision was made on the alveolar ridge of tooth #29 after anesthetizing it with Primacaine Adrenaline (Produits Dentaires Pierre Rolland, France). The surgical area was exposed using a periosteal stripper, alveolar bone was trimmed and the implant socket was prepared. Class-I bone was encountered, for which the Osstem TSIII SA drill bit is recommended after the final drill before implant placement, but it was not used. As a result, the implant did not reach the expected position at the torsional strength of 35 N. The implant with the carrier reached 0.5 mm below the bone with a torsional strength greater than 35 N through manual instrumentation. The central screw of the carrier could not be detached and fractured when dental forceps were used. The clinician tried to retrieve it using an ultrasonic scaler, but it dropped to the apical aspect of the implant (Figures 1B–3B), making it quite difficult to remove. Therefore, the clinician decided to immediately remove the implant to avoid the need for a second surgery.

Due to the high torque and fractured screw, the implant could not be removed using a wrench. Trephine bur could also not be used because of the high bone density. A fissure bur was used to remove the bone from facial aspect down to the implant apex. A vertical gingival incision was made distal to achieve adequate exposure and operational convenience. The implant was grasped using dental forceps and an attempt was made to retrieve it through rotational and rocking movements. Most of the buccal bone was removed, leaving a wall of approximately 2 mm at the implant apex. Fortunately, most of the lingual wall was preserved. A three-wall bone defect measuring about 4.7 mm × 3.9 mm × 11.4 mm was created (Figures 1B and 3B).

**Figure 4.**
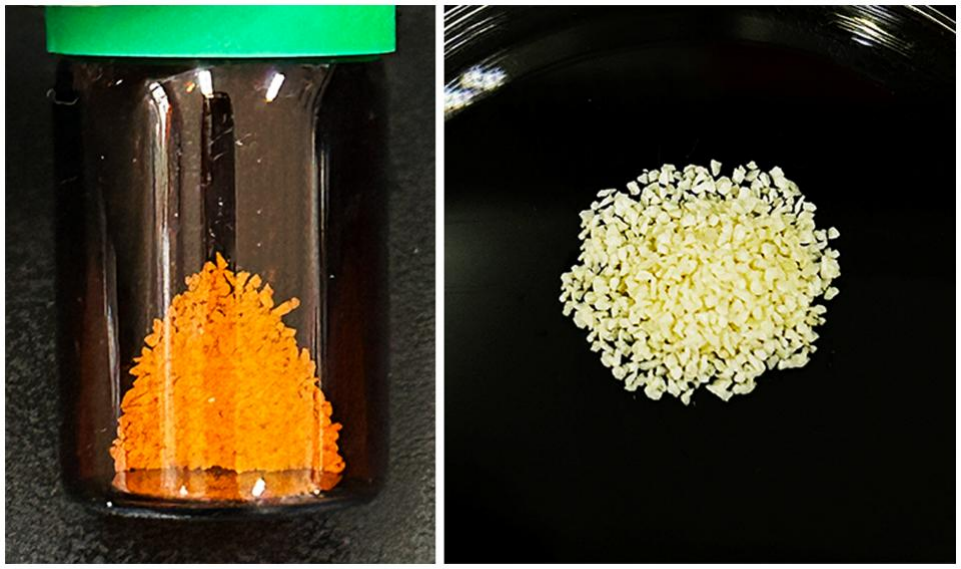
The SD-FDBA used in this case.

After implant removal, 0.5 g of SD-FDBA (particle size: 0.05–0.95 mm) mixed with the patient’s blood was used to fill the defect. The photographs of SD-FDBA are shown in Figure 4. The incision was closed with interrupted non-absorbable sutures (3-0, Hangzhou HuaWei Medical Supplies Co., Ltd., China). The patient was prescribed antibiotics postoperatively, and the sutures were removed after 10 days.

CBCT was performed at 1 week, 6 months and 15 months postoperatively to assess bone regeneration. Anatomical markers were used for standardization during image reconstruction. The images were compared with those obtained before implantation (Figures 1–3). The bone defect was marked as a red box or oval, and the mandibular neural canal was marked as a red line or dot.

The horizontal plane passed through the top of the fractured screw, approximately 23 mm above the lower border of the mandible (Figure 1). The pulp cavity of tooth #27 and the distal buccal pulp cavity of tooth #30 were marked as points 1 and 2, respectively, in this section. The sagittal plane was based on these two points, perpendicular to the horizontal plane (Figure 2). The coronal plane was defined as a plane at the midpoint between points 1 and 2, perpendicular to the sagittal plane (Figure 3).

The boundary of the bone defect was not obvious because the densities of SD-FDBA and native bone were similar. However, the defect could be visualized, particularly in the apical half (Figures 1C and 2C). The density of the bone defect increased with time, and the defect boundary remained unclear at 6 and 15 months (Figures 1–3). It indicated the trabecular hyperplasia and activity of osteoblasts and osteoclasts.

To quantitatively compare the dimensional changes due to implant extraction and grafts implantation, the height and width of the alveolar ridge were measured 1 week, 6 months and 15 months after bone grafting (Figures 1 and 3, Table 1). The percentage of remaining alveolar height or width was calculated as follows [11, 12]:

$$\begin{array}{c} \mathrm{Remaining}\;\mathrm{percentage}\;\left(\%\right)=\\ \frac{\mathrm{Alveolar}\;\mathrm{ridge}\;\mathrm{height}\;\left(\mathrm{width}\right)\;\mathrm{after}\;\mathrm{bone}\;\mathrm{grafting}}{\mathrm{Alveolar}\;\mathrm{ridge}\;\mathrm{height}\;\left(\mathrm{width}\right)\;\mathrm{before}\;\mathrm{bone}\;\mathrm{grafting}}\times 100\% \end{array}$$

**Table 1.** Alveolar ridge height and width before and after bone grafting

	Height (mm)	Remaining percentage (%)	Width (mm)	Remaining percentage (%)
Before implantation	14.3	–	6.9	–
1 week after bone grafting	13.7	95.8	8.3	120.3
6 months after bone grafting	13.1	91.6	6.6	95.7
15 months after bone grafting	13.1	91.6	6.3	91.3

The post-extraction alveolar height was about 91% of the original value. The width increased slightly because of the overfilling of bone graft, but gradually decreased to a steady level of about 91%.

Gray value ratios of the defect at different time points (Figure 3), which represent the bone density, were compared quantitatively. The control sites were chosen from the maxillary alveolar bone (site 3), while the experimental sites were chosen from the bone defect in the coronal plane (site 4). Sites 3 and 4 had equal sizes (a 3.4-mm side length square) and were located at the same place on CBCT images at different time points. For site 3, it was determined by the alveolar bone images of tooth #5, which was located between the buccal bone wall and maxillary sinus floor bone wall of tooth #5. For site 4, it was 4.6 mm above the upper margin of the mandibular nerve canal, and 5.4 mm left to the lingual bone wall of tooth #29. The relative gray value ratio was calculated using Xelis Dental (1.0.6.2 BN11, Quantitative Radiology) as follows:

$$\mathrm{Relative}\;\mathrm{gray}\;\mathrm{value}\;\mathrm{ratio}=\frac{\mathrm{Mean}\;\mathrm{gray}\;\mathrm{value}\;\mathrm{of}\;\mathrm{site}\;4}{\mathrm{Mean}\;\mathrm{gray}\;\mathrm{value}\;\mathrm{of}\;\mathrm{site}\;3}$$

A significant decrease in the gray value ratio 1 week after bone grafting was due to uneven and unconsolidated filling of the granular graft in the defect particularly the bottom half. The values increased gradually from 1 week to 15 months and approached the values before the first implantation (Table 2). This was also consistent with the gross bone mineral density changes observed (Figure 3), indicating favorable bone regeneration with SD-FDBA. During the 15-month period between bone allograft and reimplantation, the patient was not followed up regularly due to the outbreak of COVID-19.

**Table 2.** Gray value ratios of the chosen experimental and control sites before and after bone grafting

	Mean gray value	Gray value ratio
Before implantation		
Control site	361.1	4.05
Experimental site	1464	
1 week after bone grafting		
Control site	255.6	2.04
Experimental site	521	
6 months after bone grafting		
Control site	336.7	3.49
Experimental site	1176	
15 months after bone grafting		
Control site	400	3.64
Experimental site	1455	

**Figure 5.**
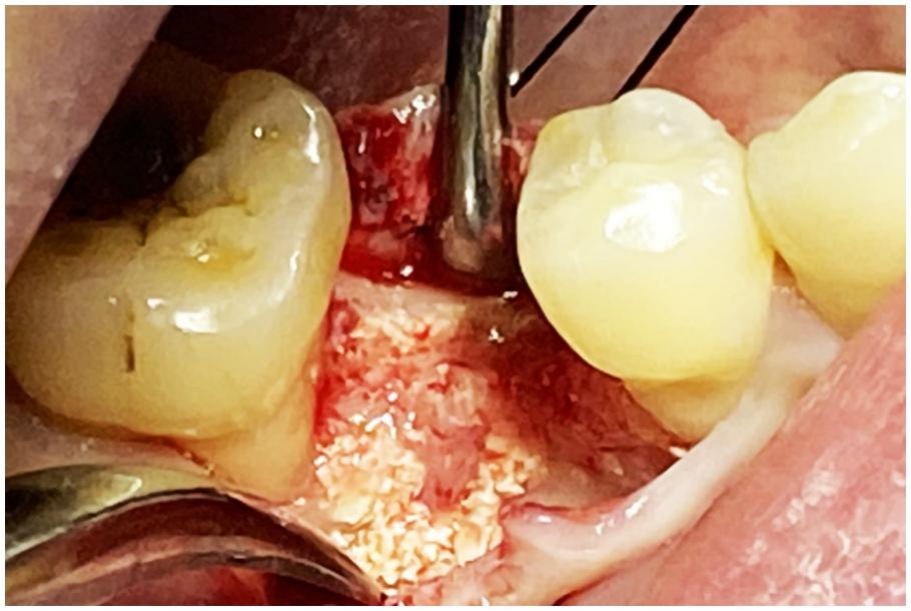
Intra-oral photographs during reimplantation.

At 15 months, the bone defect had achieved the required bone regeneration, and reimplantation was planned. When the bone defect was reopened, large quantities of the white granular bone graft were encountered (Figure 5). A small amount of the graft was not integrated, while most were completely integrated with the native bone and hard enough for implantation. The new bone bled when drilled, indicating good bone regeneration and vascularization.

A 4.0 mm in diameter and 8 mm in height Dentium implant (Superline, Republic of Korea) was placed, instead of some novel implants with immunoregulatory surface treatment [13, 14]. Compared to the first implant, the location of the implant was closer to the lingual wall where the native bone was available (Figures 1F and 3F) and a wider implant was used for the sake of stability [15]. Efforts had been made to acquire bone tissue during replantation but failed due to high bone density and limited bone quantity.

CBCT images before (15 months after bone grafting), immediately after, and 8 months after reimplantation were obtained for quantitative analysis, replacing point 2 with point 5 (distal lingual pulp cavity of tooth #30). The remaining alveolar height or width percentage was calculated as [11, 12]:

$$\begin{array}{c} \mathrm{Remaining}\;\mathrm{percentage}\;\left(\%\right)=\\ \frac{\mathrm{Alveolar}\;\mathrm{ridge}\;\mathrm{height}\;\left(\mathrm{width}\right)\;\mathrm{after}\;\mathrm{reimplantation}}{\mathrm{Alveolar}\;\mathrm{ridge}\;\mathrm{height}\;\left(\mathrm{width}\right)\;\mathrm{before}\;\mathrm{reimplantation}}\times 100\% \end{array}$$

**Figure 6.**
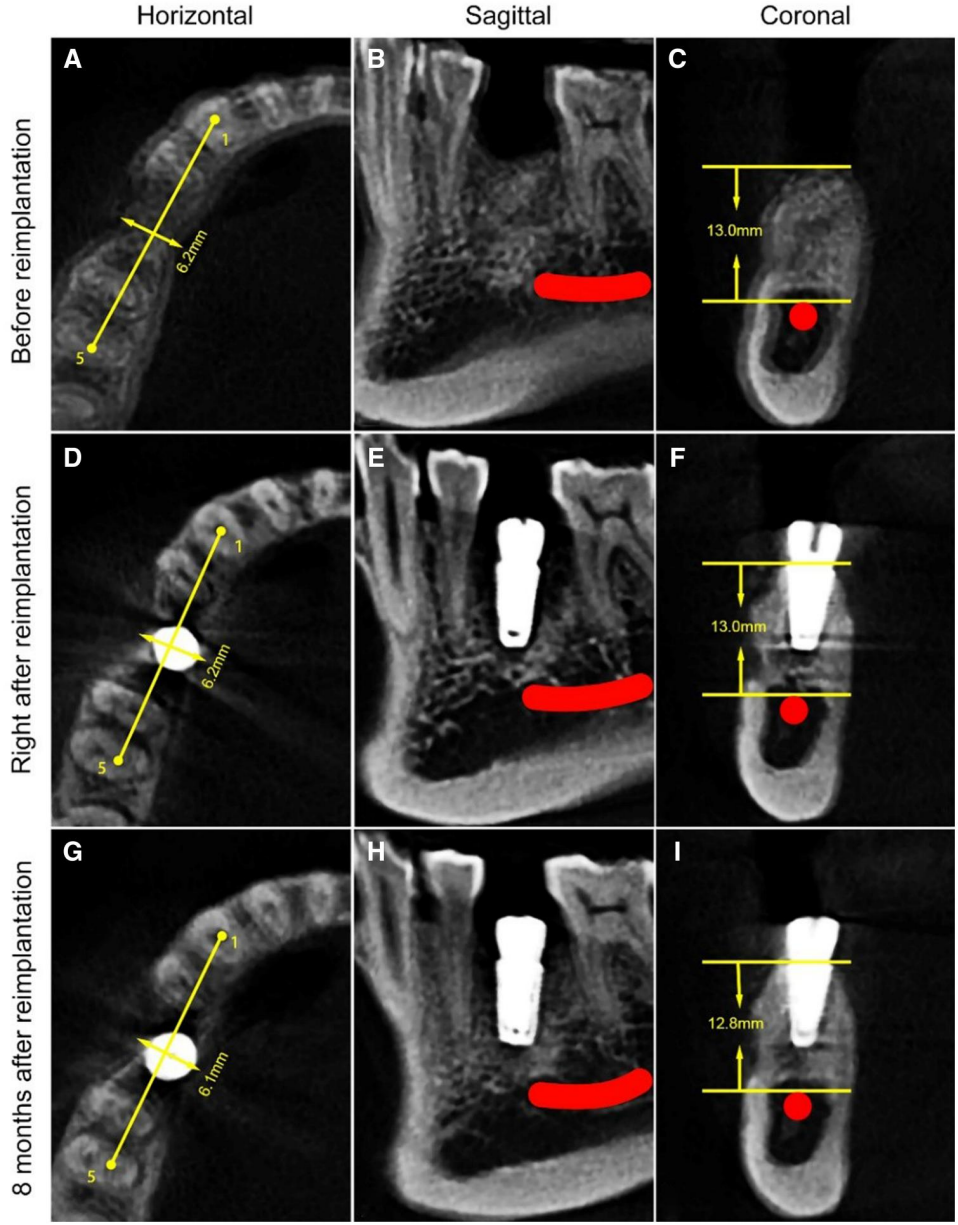
CBCT Images. (**A–C**) Before reimplantation (15 months after bone grafting), (**D–F**) immediately after reimplantation; (**G–I**) 8 months after reimplantation. Red line and dot indicate the mandibular nerve canal.

**Figure 7.**
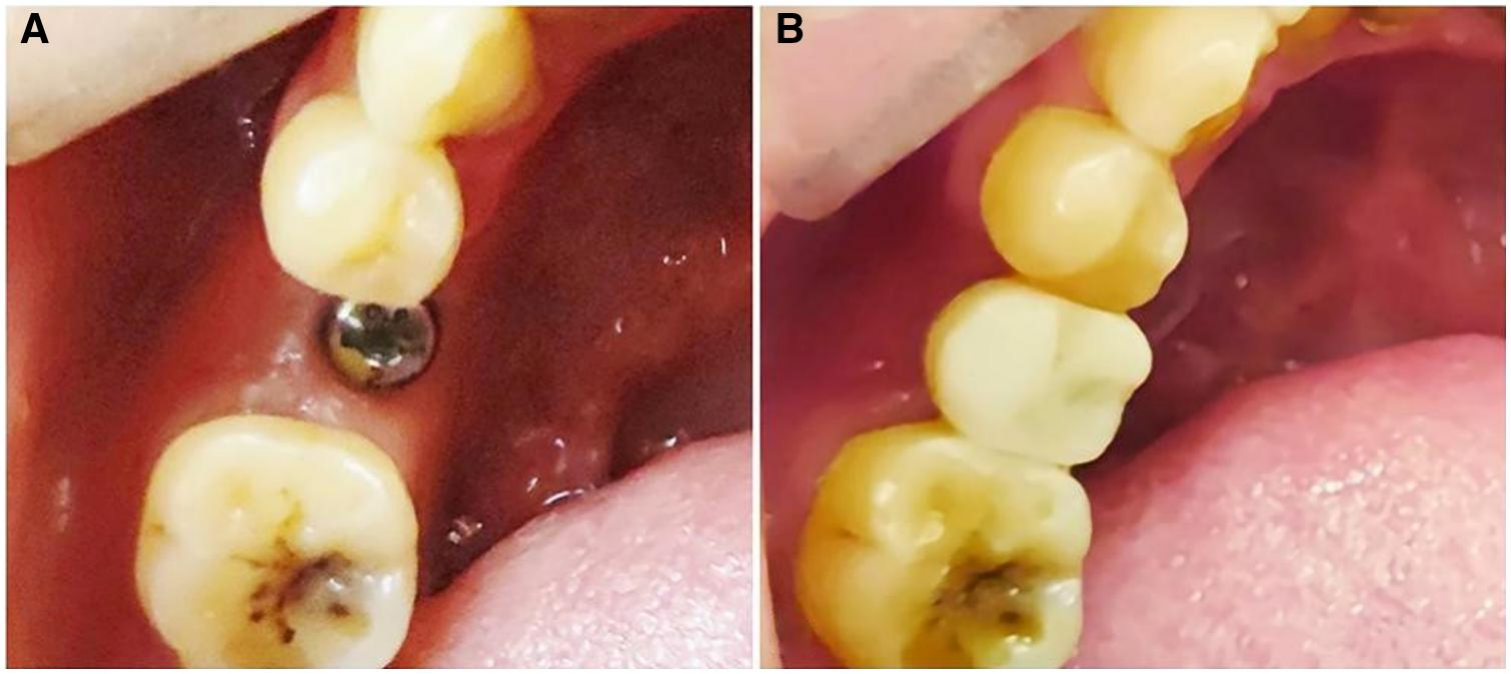
Intra-oral photographs after repair. (**A**) Implant with the healing cap and (**B**) screw-retained zirconia single crown restoration.

The bone level around the implant remained stable (Figure 6, Table 3). Digital impressions were obtained and a screw-retained zirconia crown was fabricated and fixed. A final torque of 35 N was achieved (Figure 7). The implant and prosthetic crown were in good condition in the follow-up 6 months and 1 year later.

**Table 3.** Alveolar ridge height and width before and after reimplantation

	Height (mm)	Remaining percentage (%)	Width (mm)	Remaining percentage (%)
Before reimplantation	13.0	–	6.2	–
Immediately after reimplantation	13.0	100.0	6.2	100.0
8 months after reimplantation	12.8	98.5	6.1	98.4

**Figure 8.**
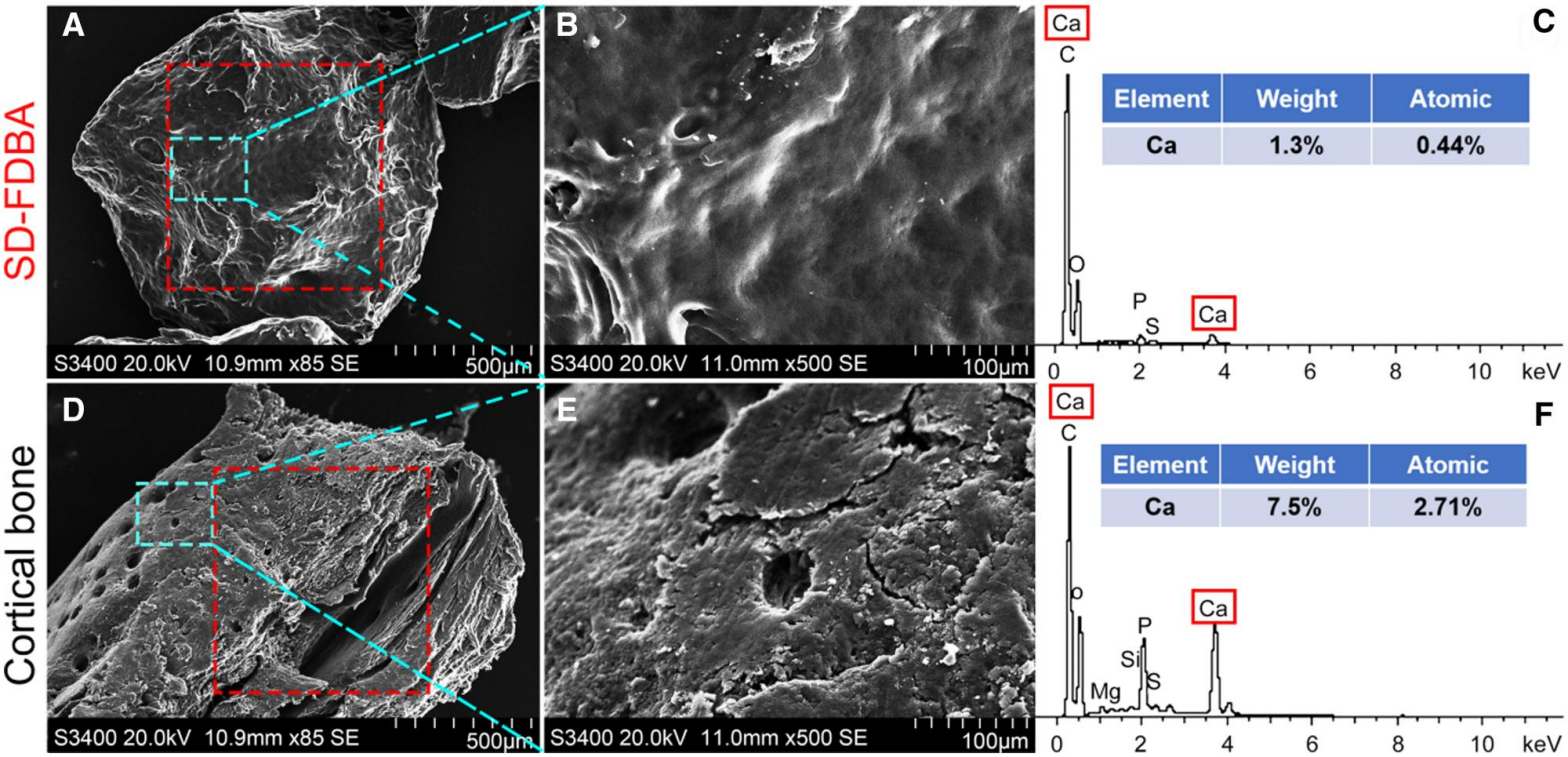
SEM Images with EDS of the SD-FDBA. (**A–C**) SD-FDBA and (**D–F**) Cortical bone.

**Figure 9.**
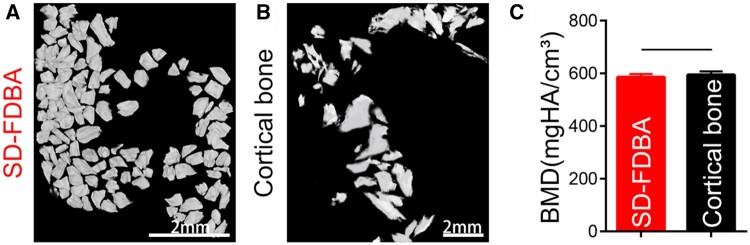
3D Reconstruction images. (**A**) SD-FDBA, (**B**) cortical bone and (**C**) BMD measurement (*P *>* *0.05).

**Figure 10.**
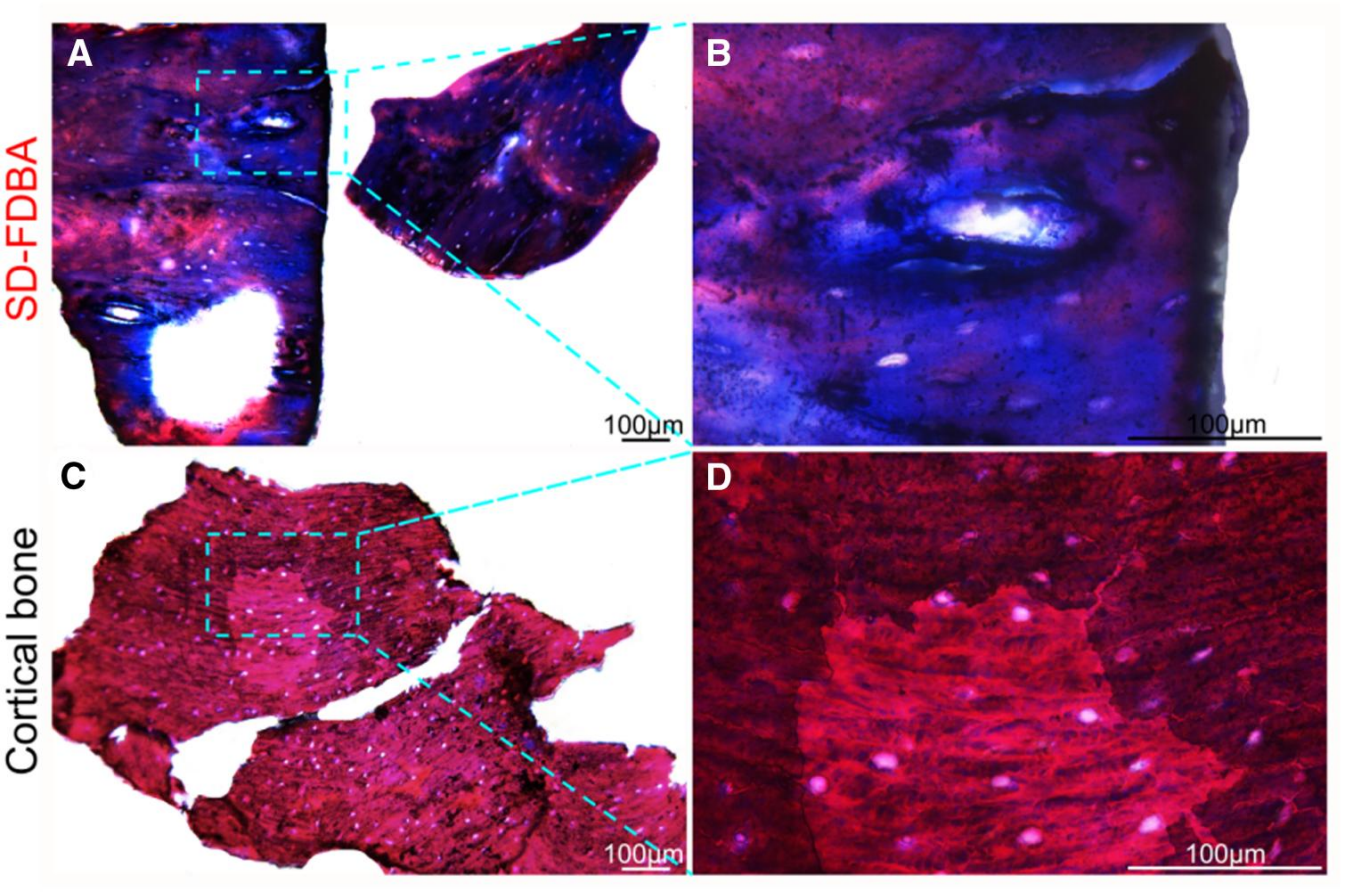
The undecalcified histological sections of SD-FDBA (masson staining). (**A–B**) SD-FDBA and (**C–D**) cortical bone.

**Figure 11.**
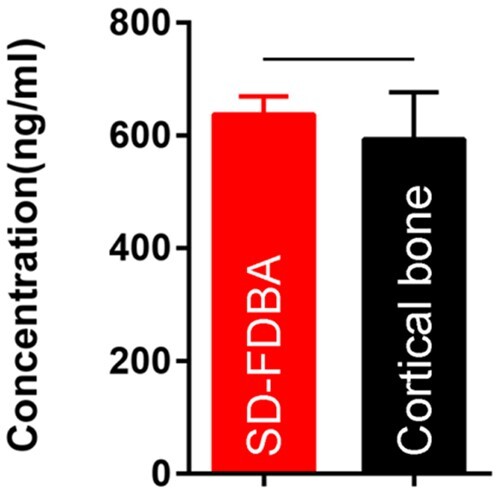
BMP-2 content by elisa. (*P *>* *0.05).

Many important information of this SD-FDBA such as the content of BMP-2 and the process of demineralization was not provided by the manufacturer. Therefore, it was thoroughly characterized including its structure and composition to explore the reasons for its excellent osteogenic properties. Under an SEM, it has a rough and laminated structure with several big pores. Compared with fresh human cortical bone, its porous structure is partly collapsed and the content of calcium on the surface is decreased (Figure 8), which may be due to the processing of the material. As presented by micro-CT images, the mineralization degree of SD-FDBA is similar to cortical bone (*P *>* *0.05, Figure 9). However, Masson staining of the undecalcified SD-FDBA sample confirmed its partly demineralized condition, especially the surfaces (Figure 10). Moreover, the BMP-2 level of SD-FDBA was similar to that of the cortical bone (*P *>* *0.05, Figure 11).

## Discussion and literature review

### Causes of dental implant failure

Failures are classified into early and late failures based on the timing relative to abutment connection and functional loading. Common causes of early failures include poor bone quality, overheating of bone during socket preparation, lack of primary stability due to over-preparation of the implant site and infections [16], leading to failed osseointegration. Late failures include the failure of established osseointegration or implant function. They are often related to patient’s condition (history of radiation therapy, bruxism, periodontitis or smoking), clinical parameters (implant location) and prosthetic type (fixed or removable prostheses) [17].

Failures can also be classified as biological, caused by soft and hard tissue resorption, and mechanical, caused by the fracture of implant, screw or supra-structure due to inappropriate implantation or loading form [15]. In mechanical failures, the implant is often immobile and remains at least partially osseointegrated in the apical region. Implant removal is challenging in these cases and requires an invasive approach which increases the risk of pain and trauma to the adjacent structures.

Based on these definitions, the failure in the present case was early mechanical failure. Implant position and stability were good, and it may have been preserved if the fractured screw could be retrieved or the implant could be restored despite the fractured screw.

### Management of dental implant with fractured screw

Carrier screws are not an essential accessory of the implant system, and several systems, such as Nobel and Dentium, do not have carrier screws. However, the abutment screw is an indispensable prosthetic component that connects the abutment with the implant. The incidence of carrier and abutment screw fracture is not high, but the retrieval of fractured screws is very difficult.

The initial step in the management of fractured screws is to obtain a detailed history and perform a thorough clinical examination. Raju et al. systematically reviewed the management of fractured abutment screws to develop a logical sequence for management [18]. They reported that the harm to implants resulting from the removal of abutment screw fragments ranges from mild to severe regardless of the technique used. Generally, a rigid instrument, such as a scaler, sickle explorer, or endodontic explorer, is combined with ultrasonic oscillations to rotate the screw fragment in a counter-clockwise direction until it loosens. If this fails, burs or drills mounted on a handpiece and rotated in a counter-clockwise direction can be used to unwind the fragment. A retrieval kit may also be used. A hole can be made in the fractured screw to unwind it by engaging a drill [18]. Abutment screws modified with a prefabricated access hole are easier to retrieve [19].

If the implant’s internal screw threads are significantly damaged, or the fractured screw cannot be retrieved, the implant may need to be removed. The fabrication of a custom cast post and core is the final option in case the patient cannot be persuaded to implant removal [18]. However, this weakens the implant and produces excessive heat during the cutting of the fractured screw [18]. Screws fractured in the apical portion are even more difficult to retrieve and pose a greater risk to implant survival. Therefore, it is better to take measures to avoid screw fractures.

### Implant removal

The decision to remove an implant is based on clinical and radiographic assessment. Pain, vertical mobility and progressive bone loss are common reasons for implant removal [20]. These conditions often make it easier to remove the implant. Screw fracture is a rare cause of mechanical implant failure. Immediate removal is not necessary because the implant usually has a high torque. In the present case, however, the implant was removed immediately after failure to avoid the trauma of a second surgery. However, immediate removal resulted in significant bone loss, which required grafting and delayed reimplantation.

Local and systemic factors, including pre-load, infections and hyperglycemia, can negatively influence the secondary stability and osseointegration despite good primary stability. In such cases, the non-osseointegrated implants can be easily removed with less trauma. Implant stability is the lowest during the changeover from primary stability, achieved at the time of implant placement, to secondary stability achieved by new bone deposition [21]. Primary mechanical stability gradually gives way to biological stability resulting from a series of cellular and molecular events at about 4 weeks after implant placement [22]. Trauma associated with implant removal is reduced if the implant is not osseointegrated or is removed when the torque is lowest.

A variety of techniques can be used to remove implants, including the counter-torque technique, bur-forceps technique, trephine technique, piezo surgery and laser surgery [23–27]. A comparison of different implant removal methods and their advantages, disadvantages and indications are shown in Table 4. Removal of strongly osseointegrated implants requires excessive bone removal, which decreases the bone volume for reimplantation. In the present case, a method similar to the bur-forceps technique was used, which led to a large bone defect.

**Table 4.** Implant removal methods [23–27]

Technique	Advantages	Disadvantages	Indications
Counter-torque ratchet	Less traumatic	Low success rate. Need for special instruments or kits.	Extraction inserts can be screwed into the implant. Implant with a low torque.
Trephine technique	Simple to use with a guiding pin.	Aggressive. Risk of osteonecrosis. Impaired bone regeneration. Complicated immediate implant placement.	Failed counter-torque ratchet method. Bone-level implants with adequate bone and distance from adjacent structures.
High speed bur or Bur-forceps technique	Reliable and predictable. High success rate. Efficient.	Drilling noise. Uncontrolled forces. Time consuming. Traumatic and invasive.	Failed counter-torque ratchet method. Failing implants without a gap with the neighboring tooth/implant.
Piezo surgery	High precision. Less damage to soft tissues. Local hemostasis and improved visibility	Costly and time consuming. Not suitable for patients with pacemakers. Requires special equipment.	Failed counter-torque ratchet method. Cuts shallow bone well. Exclude patients with a pacemaker.
Laser surgery	Less invasive and traumatic. Limited thermal injury. Good visualization.	Excessive hand pressure on the optical tip may cause fracture. Costly and time consuming. Requires special equipment.	Failed counter-torque ratchet method. Exclude patients that have a propensity for poor wound healing. Exclude patients that are immuno-compromised or had previous jaw radiotherapy.
Electro-surgery	Conservative	Costly and time consuming. Requires special equipment. Limited clinical evidence	Failed counter-torque ratchet method.

### Management of bone defect

Bone defects after the removal of failed implants present a therapeutic challenge. The shape of the bone defect in the present case was similar to a tooth extraction socket. However, trauma to the residual bone and osteoblasts is greater in implant extractions than in tooth extractions, which negatively influences bone regeneration. A favorable factor in the present case was the presence of adequate soft tissue to cover and seal the bone defect.

Reimplantation can be performed immediately after implant removal if primary stability can be achieved, or the defect can be allowed to heal followed by delayed reimplantation. Immediate implantation requires fewer surgeries and improves patient satisfaction. However, this may not be possible in cases with compromised primary stability, poor osteogenic cell function and excessive inflammatory response [28]. A meta-analysis demonstrated an increased risk of implant failure with the immediate implantation protocol [28]. In the present case, the trauma and heat generated during implant extraction were likely to delay or hinder osseointegration. Therefore, ARP was performed to maintain the horizontal and vertical alveolar ridge form, followed by delayed reimplantation.

The current ARP techniques utilize bone grafts, bone substitutes, barrier membranes and biological products, either alone or in combination [29–33]. The advent of newer biomaterials has ignited an interest in the best technique for ARP, but there is currently no consensus. Xenografts are the best option for ARP after tooth extraction because their slow degradation rate improves dimensional preservation [34]. It has been demonstrated that autografts present the highest percentage of new bone formation, followed by synthetic grafts, xenografts and allografts. However, there were no significant differences in the percentage of new bone formation between any two graft types [35]. A combination of graft materials may enhance their functionality and broaden the indications.

Barrier membranes can be used to prevent non-osteogenic cells, such as gingival epithelium and connective tissue, from entering the defect. Barrier membranes are not essential for ARP, but improve the outcomes when combined with bone grafts [36]. Some resorbable membranes, such as Bio-Gide, require complete coverage for ARP. In contrast, Mucograft Seal, a porcine-derived collagen membrane, can be used to achieve primary closure in ARP in combination with soft tissue management techniques, such as full-thickness envelope flaps, free and pedicled grafts, and releasing incisions. In the present case, the bone defect was grafted with an allograft and sealed using gingival sutures without a barrier membrane.

Autologous platelet concentrates are blood-derived bioactive products containing fibrin and growth factors that can be used in ARP [37]. Based on their leukocyte and fibrin content, they are categorized into pure platelet-rich plasma, leukocyte- and platelet-rich plasma, pure platelet-rich fibrin (P-PRF) and leukocyte- and platelet-rich fibrin. Their effects on soft tissue healing are well-established but remain controversial in bone healing [38].

Bone grafts and membranes may contain growth factors, such as bone morphogenetic protein, transforming growth factor, platelet-derived growth factor (PDGF), vascular endothelial growth factor and insulin-like growth factor, which can aid cell recruitment, cell differentiation and osteogenesis [39–41]. Furthermore, stem cells and tissue engineering are the focus of current research and may improve future treatment.

### Allografts for bone augmentation

The properties of allografts depend on the conditions, such as age, of the donor. There are considerable variations among commercially available allografts obtained from different bone banks and different samples from the same tissue bank. Allograft properties are also influenced by the processing and sterilization methods, including solvent dehydration, freeze-drying and gamma irradiation [42]. We previously proposed the use of polyvinylpyrrolidone-iodine solution for sterilization and preservation of allografts for improved mechanical properties and osteogenesis [43].

In combination with other materials, allografts can optimize the environment for bone regeneration. When mixed with autogenous bone, they can reliably achieve vertical ridge augmentation to obtain a high percentage of vital mineralized tissue [44]. In combination with concentrated growth factor membranes, DFDBA can increase the height, depth and mesiodistal width of periodontal intrabony defects [45]. The combination of P-PRF and DFDBA, grafted between the implant body and socket wall in immediate implant placement, significantly reduces bone resorption and maintains the buccolingual dimension of the bone [46]. Recombinant human PDGF-BB combined with FDBA and a resorbable barrier membrane significantly improves clinical attachment levels and provides a bone-like fill radiographically and at reentry [47].

The combination of FDBA and DFDBA provides an ideal combination of properties from both components. The 70% FDBA/30% DFDBA combination was found to achieve greater vital bone compared to 100% FDBA (36.2% vs. 24.7%) at 18–20 weeks after ridge preservation [9]. Longer healing times lead to greater vital bone formation and less residual graft at the time of implant placement [10]. In ARP after tooth extraction, the residual allograft material may remain in the socket for up to 12 months [48]. In the present case, the healing time was 15 months. Surface demineralization gives SD-FDBA the advantages of both DFDBA and FDBA. The demineralized part is osteoinductive, while the mineralized part is osteoconductive. With the demineralized part, the initial demineralization process is not required, and the soluble osteoinductive proteins such as BMP-2 are able to function immediately after implantation. A beneficial prolongation of osteoinductive protein release is expected in the mineralized part due to a prolonged osteoclastic breakdown. Therefore, SD-FDBA has excellent osteogenic properties.

### Prognosis and long-term stability of reimplantation

It is generally believed that dental implants placed in sites of previous implant failure have lower survival rates compared to the initial implant, and that the survival rate decreases with each successive reimplantation. The survival rate for a second placement was found to be 93%, while it was 85% for a third placement in the same site [49, 50].

The quality and quantity of bone at the recipient site are critical to implant success and long-term survival. Bone quality, evaluated as bone density and the distribution of cortical-medullary bone, significantly influences the mean marginal bone loss. The new bone has poorer mineralization maturity, indicated by a lower bone density. Furthermore, residual graft material may interfere with osseointegration. The difficulties in reimplantation at a failed site include primary stability, osseointegration with the new bone, and long-term stability of marginal bone. However, it has been reported that Bio-Oss does not affect bone remodeling and maturation around the dental implant [51]. Placement of Bio-Oss in the extraction socket increased the bone-implant ratio and mineralized bone area, which may be related to the mechanism of bone remodeling and maturation [51].

Implant size type and surface treatment [13, 14] also influence osseointegration and survival in reimplantation cases. Novel implants with functional surface treatment have the potential to improve the clinical outcomes of osteoporotic patients [52]. The choice of implant should be based on the local tissue condition, available bone volume, and the anticipated bite force. Implant length and diameter affect osseointegration, the initial osseointegrated area and its mechanical strength. Generally, the same length and diameter are used during reimplantation as the primary implant. A longer or wider implant may be used in some cases to provide better primary stability. In the present case, the implant was shorter than the first implant because of the decreased vertical bone height. The diameter was slightly wider to ensure adequate bone-to-implant contact and mechanical support for the future prosthesis. A conservative embedded healing approach was used, which has a higher success rate than open healing.

Long-term implant survival also depends on the success of the prosthesis. More than 10% of the crowns have to be replaced within 10 years after restoration due to biological or technical reasons [53]. Common reasons for technical failures in single implant-retained crowns include fractured or loosened abutment/prosthetic screws, poor cemented-crown retention, and chipped or fractured ceramic.

Although long-term results of reimplantation require further investigation, improved patient care, particularly in the psychological aspects, is important for implant success and long-term stability.

### Ethics approval and consent to participate

The study protocol was approved by the Ethics Committee of Nanjing Medical University (PJ2021-152-001). The patient was treated in accordance with the Declaration of Helsinki. Informed consent of the patient was obtained after explaining the clinical procedures, risks and benefits and clarifying all questions raised by the patient.

## Supplementary Material

rbad102_Supplementary_Data
