# Supplementary material for: Surface demineralized freeze-dried bone allograft followed by reimplantation in a failed mandibular dental implant
Source: Regen Biomater. 2023 Nov 17;11:rbad102. doi: 10.1093/rb/rbad102 (PMC10761198; doi:10.1093/rb/rbad102)
Supplement: rbad102_Supplementary_Data [file rbad102_supplementary_data.docx]

Supplementary Material For

**Surface demineralized freeze-dried** **bone allograft followed by reimplantation in a failed mandibular dental implant**

Jing Zhang^1,2#^, Jie Wang^1,2#^, Jiayi You^1,2^, Xuan Qin^1,2^, Huimin Chen^3^, Xiantong Hu^4,5,6^, Yantao Zhao^4,5,6*^, Yang Xia^1,2*^

^1^ Jiangsu Key Laboratory of Oral Diseases, Nanjing Medical University, Nanjing, Jiangsu 210029, PR China;

^2^ Jiangsu Province Engineering Research Center of Stomatological Translational Medicine, Nanjing Medical University, Nanjing, Jiangsu 210029, PR China;

^3^ Department of Restorative Dental Sciences, Faculty of Dentistry, The University of Hong Kong, Hong Kong SAR, PR China;

^4^ Senior Department of Orthopedics, the Fourth Medical Center of PLA General Hospital, Beijing 100048, PR China;

^5^ Beijing Engineering Research Center of Orthopedics Implants, Beijing 100048, PR China;

^6^ State Key Laboratory of Military Stomatology, Xi'an 710032, PR China.

# These authors contribute equally to this work.

Correspondence:

Yang Xia

Jiangsu Key Laboratory of Oral Diseases, Nanjing Medical University, Nanjing, Jiangsu 210029, China

Tel.: 025-86516414

Fax: +86-25-86516414

Email: [xiayang@njmu.edu.cn](mailto:xiayang@njmu.edu.cn)

Yantao Zhao

Institute of Orthopedics, Fourth Medical Center of the General Hospital of CPLA, Beijing 100048, China

Tel.: 010-66848865

Fax: +86-010-66848865

Email: [45828016@qq.com](mailto:45828016@qq.com)

**1. Collection of fresh human cortical bone.**

Fresh human cortical bone was obtained from patients aged 18-25 undergoing extraction of the impacted third molar [1,2] at the [oral and maxillofacial surgery](javascript:;) department of the Affiliated Stomatological Hospital of Nanjing Medical University. Informed consent was given by the donor, and the procedure was approved by the Ethics Committee of Nanjing Medical University (PJ2021-152-001).

**2. Experimental detail for micro-CT.**

SD-FDBA were scanned by micro-CT (SkyScan 1176) under the conditions: 100 kV, 98 mA, resolution = 9 μm, and single 360-degree scan [3,4]. Two-dimensional (2D) images were used to generate 3D reconstructions using CT vox 3.3 [5]. Bone mineral density (BMD) was quantified with the CTAn software. All data were expressed as means ± SD. Statistical analyses were performed using unpaired t-tests by GraphPad Prism 6.01 (San Diego, United States) [3,6]. Significance level was set at **p* < 0.05 [4,7].

**3. Experimental detail for undecalcified histological sections**

Fresh human cortical bone and SD-FDBA were embedded in methyl methacrylate resin under vacuum at room temperature after drying [5]. 10 μm thick sections were ground and polished by EXAKT precision cutting (EXAKT 300CP, **Germany**) and grinding system (EXAKT 400CS/AW, **Germany**). The slice was subjected to Masson staining using Masson's Trichrome Stain Kit (Solarbio, China). The samples were observed with positive fluorescence microscope (Leica DM6, **Germany**) and the images were taken with a digital camera (Leica DMC5400, **Germany**) using image acquisition software (LAS V4.2) [3].

**4. Experimental detail for BMP-2 mesurement.**

BMP-2 concentration of SD-FDBA was tested by enzyme-linked immunosorbent assay (ELISA, ab270552, Abcam, United States) [8], using fresh human cortical bone as control. Briefly, the samples were soaked in 1 ml ultra-pure water for 1 week, 50 μL supernatant was pipetted in duplicate into the wells of a precoated ELISA plate [9]. 50 μL antibody solution (420ng/ml) was added into each well and incubated for 60 min at room temperature on a plate shaker. After adding 100 μL stopping solution, the absorbance was measured at 450 nm by a spectrophotometric enzyme marker (Infinite M200pro, Tecan). Concentrations of BMP-2 were calculated according to the standard curve.

References

1. Chen YW, Lee CT, Hum L, Chuang SK. Effect of flap design on periodontal healing after impacted third molar extraction: a systematic review and meta-analysis. *Int J Oral Maxillofac Surg* **2017**;46:363-72.

2. Fan W, Crawford R, Xiao Y. Enhancing in vivo vascularized bone formation by cobalt chloride-treated bone marrow stromal cells in a tissue engineered periosteum model. *Biomaterials* **2010**;31:3580-9.

3. Tournier P, Guicheux J, Paré A, Veziers J, Barbeito A, Bardonnet R, Corre P, Geoffroy V, Weiss P, Gaudin A. An Extrudable Partially Demineralized Allogeneic Bone Paste Exhibits a Similar Bone Healing Capacity as the "Gold Standard" Bone Graft. *Front Bioeng Biotechnol* **2021**;9:658853.

4. Zou W, Li X, Li N, Guo T, Cai Y, Yang X, Liang J, Sun Y, Fan Y. A comparative study of autogenous, allograft and artificial bone substitutes on bone regeneration and immunotoxicity in rat femur defect model. *Regen Biomater* **2021**;8:rbaa040.

5. Liu H, Li W, Liu YS, Zhou YS. Bone micro-architectural analysis of mandible and tibia in ovariectomised rats: A quantitative structural comparison between undecalcified histological sections and micro-CT. *Bone Joint Res* **2016**;5:253-62.

6. Bertassoli BM, Silva GAB, Albergaria JD, Jorge EC. In vitro analysis of the influence of mineralized and EDTA-demineralized allogenous bone on the viability and differentiation of osteoblasts and dental pulp stem cells. *Cell Tissue Bank* **2020**;21:479-93.

7. Ullah I, Abu-Dawud R, Busch JF, Rabien A, Erguen B, Fischer I, Reinke P, Kurtz A. VEGF - Supplemented extracellular matrix is sufficient to induce endothelial differentiation of human iPSC. *Biomaterials* **2019**;216:119283.

8. E L, Lu R, Sun J, Li H, Xu W, Xing H, Wang X, Cheng T, Zhang S, Ma X, Zhang R, Liu H. Microenvironment Influences on Human Umbilical Cord Mesenchymal Stem Cell-Based Bone Regeneration. *Stem Cells Int* **2021**;2021:4465022.

9. Liu F, Wang X, Zheng B, Li D, Chen C, Lee IS, Zhong J, Li D, Liu Y. USF2 enhances the osteogenic differentiation of PDLCs by promoting ATF4 transcriptional activities. *J Periodontal Res* **2020**;55:68-76.
